# Development of an optimized protocol for protoplast-to-plant regeneration of selected varieties of *Brassica oleracea* L.

**DOI:** 10.1186/s12870-024-06005-4

**Published:** 2024-12-30

**Authors:** Katarzyna Stelmach-Wityk, Kamil Szymonik, Ewa Grzebelus, Agnieszka Kiełkowska

**Affiliations:** Department of Plant Biology and Biotechnology, Faculty of Biotechnology and Horticulture, University of Agriculturein Krakow, Mickiewicza 21, Krakow, 31-120 Poland

**Keywords:** Cabbage, Collard, Protoplast cultures, Plant regeneration

## Abstract

**Background:**

*Brassica oleracea* L. is a key plant in the *Brassicaceae* family, known for popular vegetables like cabbage, broccoli, kale and collard. Collard (*B. oleracea* var. *viridis*) is a non-heading leafy vegetable grown in urban farms and community gardens in the United States and Europe. Improving collard and other *Brassica* germplasm can benefit from both traditional and new plant breeding technologies (NPBTs), such as CRISPR-Cas mediated transformation. An efficient transformation or protoplast fusion can only be achieved with a robust and reproducible protocol for protoplast-to-plant regeneration. This research focuses on optimizing in vitro culture conditions to enhance cell divisions, microcallus formation, and the regeneration of shoots and roots in four *Brassica oleracea* varieties, including collard.

**Results:**

The protocol of protoplast release, purification and immobilization was optimized to obtain a suitable number and quality of protoplasts from seven cultivars of *B. oleracea*. The protoplast isolation efficiency after digestion of young leaves in optimized enzyme solution reached on average 2.5 × 10^6^ of cells per gram of fresh weight. Protoplasts were embedded in thin alginate layers and subjected to culture in three different media. Protoplasts of all studied cultivars were viable (88.2%), underwent cell wall resynthesis and re-entered mitotic divisions in the 5th day of culture. After 30 days of culture, protoplast-derived cells of all the tested cultivars formed microcallus. Six cultivars regenerated shoots, although the shoot formation efficiency strongly depended on the genotype and composition of the regeneration medium. The regeneration medium supplemented with 1 mg l^−1^ of NAA, 1 mg l^−1^ of 2iP, 0.02 mg l^−1^ GA_3_ and with 2% of mannitol showed the highest shoot formation efficiency for five cultivars of *B. oleracea*.

**Conclusions:**

The results of this research have led to the development of a robust protoplast-to-plant regeneration protocol for four varieties of *B. oleracea* that could be exploited as a tool for production of transformants and somatic hybrids. Furthermore, we present the first successful regeneration of protoplast-derived plants of collard, an overlooked but valuable variety of *Brassica oleracea*.

**Supplementary Information:**

The online version contains supplementary material available at 10.1186/s12870-024-06005-4.

## Background

*Brassica oleracea* L. is a significant species within the *Brassicaceae* family due to its various uses and characteristics. The species has high economic and nutritional value, and includes several popular and commercially cultivated vegetables such as cabbage, broccoli, cauliflower, Brussels sprouts, kale, kohlrabi, and Savoy cabbage. *Brassica oleracea* also plays a vital role in advancing plant breeding and biotechnology by serving as a valuable genetic reservoir for creating new *Brassica* varieties with enhanced attributes such as disease resistance and yield capacity [1].

*Brassica oleracea* var. *viridis*, known as collard, might not only provide such a resource but also could benefit from other *Brassica* genetic resources. This leafy vegetable is frequently cultivated in British Isles, Portugal, Spain, Italy and the United States. Results of genetic studies indicate that cabbage (*B. oleracea* var. *capitata*) is likely the closest relative to collard [2, 3]. Over the years the genetic diversity of collard has been depleted due to the widespread use of commercial hybrids [4]. Moreover, the majority of modern cultivars are prone to fusarium yellow and black rot, which can cause serious damage when grown in warm, infested soil [5]. Fanhram et al. [6] attempted to exploit a close relationship between fusarium yellow-resistant cabbage and collard to produce hybrids and aid the development of new collard varieties with improved resistance. Although new hybrids were produced and exhibited more collard-like traits, they were not subjected to field tests in infested soil [6]. The improvement of agronomically important traits in collard could greatly benefit from using biotechnological methods like somatic hybridization or genetic transformation, both stable and transient. In fact, somatic hybridization has already proven effective for *Brassica* species in introducing genetic variability and transferring desirable traits, such as resistance to bacterial and fungal diseases or cytoplasmic male sterility [7–11]. While the potential of somatic hybridization to create new varieties may still be limited, it remains an important tool for improving multigenic traits in plant breeding.

Genetic transformation in *Brassica* has been reported in several studies, with the use of various types of explants, such as hypocotyls, cotyledons and peduncles [12–15]. More recently, protoplasts are gaining more attention as a new type of explant suitable for genome editing through transient transformation, and were also utilized in studies on genome editing in cabbage [16, 17]. Stajič et al. [16] compared two commonly used transient expression methods (protoplast transfection and agrofiltration) for genome editing in red cabbage. Both methods provide a valuable tool for testing new CRISPR/Cas9 constructs, whereas protoplast transfection proved to be more suitable for cabbage when regeneration is required.

The use of protoplasts has the potential to speed up the development of new plant breeding technologies. To successfully employ protoplast cultures and manipulations on protoplasts in practice, several key requirements need to be met. These include: (1) an efficient and consistent isolation of large quantities of highly viable protoplasts, (2) efficient methods for obtaining and culturing viable cells, and (3) the establishment of reproducible strategies for the protoplast-to-plant regeneration [18]. Each step has to be carefully tailored to the species, subspecies, or even particular genotypes of interest. A detailed analysis of protoplast culture in *B. oleracea* revealed that the genetic makeup plays a crucial role in determining the widespread application of protoplast techniques in the advancement of breeding and biotechnology in this plant species [19–23].

While previous studies have outlined various approaches for regenerating plants from protoplasts in *Brassica* species [24–27], these methods are often not easily reproducible, especially in *Brassica oleracea*. Enhancing methods for regenerating protoplasts from various *B. oleracea* varieties is crucial for progressing biotechnological advancements of current genetic resources. The main objective of this study is to optimize in vitro culture conditions to enhance cell divisions, microcallus formation, and the regeneration of shoots and roots in broad spectrum of *Brassica oleracea* (four varieties, seven cultivars). Furthermore, we present the first successful protoplast-to-plant regeneration of collard, an unacknowledged and overlooked variety of *Brassica oleracea*, showing significant potential for broadening the genetic diversity within *Brassica* species.

## Methods

### Plant material

As a protoplast source, seven cultivars of *Brassica oleracea* L. have been used (Table 1).

**Table 1:** Seed source of *Brassica* cultivars used for protoplast cultures

Species	Common name	Cultivar	Seed source
*Brassica oleracea* var*. capitata* f*. rubra*	red cabbage	Haco	PlantiCo Zielonki Sp. z.o.o., Poland
		Kalibos	PlantiCo Zielonki Sp. z.o.o., Poland
*Brassica oleracea* var*. gemmifera*	Brussels sprout	Casiopea	PlantiCo Zielonki Sp. z.o.o., Poland
		Red	PlantiCo Zielonki Sp. z.o.o., Poland
*Brassica oleracea* var. *sabellica*	kale	Kapral	PlantiCo Zielonki Sp. z.o.o., Poland
		Scarlet	PlantiCo Zielonki Sp. z.o.o., Poland
*Brassica oleracea var. viridis*	collard	Vates	Sustainable Seed Company, USA

Protoplasts were isolated from young plants germinated from seeds in in vitro conditions. For this purpose, seeds of donor cultivars were surface disinfected in 70% (*v/v*) ethanol for 2 min., 10% (*w/v*) chloramine T (Biochemie Poland, Poland) for 20 min., and washed three times with sterile distilled water (5 min. each) and air dried. Seeds were placed in sterile 500 ml plastic culture boxes (Pakler Lerka, Poland) containing approx. 80 ml of MS20 medium (Table 2) and maintained at 22 ± 2 °C with 16-h photoperiod and light intensity of 40 μmol m^−2^ s^−1^ (fluorescent lamps Sylvania Gro-lux T8, USA).

**Table 2:** Solutions and media used for the protoplast isolation and culture, callus culture and plant regeneration of selected cultivars of *Brassica olearacea* L

Solution/ medium name	Solution/ medium composition	Application	Storage conditions
MS20	MS micro- and macroelements including vitamins [28] (Duchefa Biochemie, The Netherlands), 20 g l^−1^ sucrose (POCH, PL), 0.28% (*w/v*) Gelrite (Duchefa Biochemie); pH 5.8; autoclaved	seed germination and donor plant growth	RT
PSII	0.5 M mannitol (Merck); pH 5.6; autoclaved	plasmolysis	RT
enzyme solution (ESC) [29]	0.5% (*w/v*) cellulase Onozuka R-10 (Duchefa Biochemie), 0.1% (*w/v*) pectolyase Y-23 (Duchefa Biochemie), 5 mM 2-(N-morpholino) ethanesulfonic acid (MES; Merck), 27 mM calcium chloride (POCH, Poland), 0.4 M mannitol (Merck); pH 5.8; filtered (0.22 µm membrane)	cell wall digestion	4 °C, dark
sucrose/MES	0.5 M sucrose (POCH), 1 mM MES (Merck); pH 5.8; autoclaved	protoplast separation and purification	RT
W5 [30]	154 mM sodium chloride (POCH), 125 mM calcium chloride dihydrate (POCH), 5 mM potassium chloride (POCH), 5 mM glucose (POCH); pH 5.8; autoclaved	protoplast purification	RT
CPPO1	macro- and microelements, and organic acids according to Kao and Michayluk [31] (Duchefa Biochemie), vitamins according to Gamborg B5 medium [32], 0.4 M glucose (POCH), 250 mg l^−1^ casein enzymatic hydrolysate (Merck), 0.1 mg l^−1^ 2,4-dichlorophenoxyacetic acid (2,4-D; Merck), 0.5 mg l^−1^ 1-naphthaleneacetic acid (NAA; Merck) and 0.5 mg l^−1^ 6-benzylaminopurine (BA; Merck); pH 5.6; filtered (0.22 µm membrane)	protoplast culture	4 °C, dark
Bras4	macro- and microelements, and organic acids according to Kao and Michayluk [31] (Duchefa Biochemie), vitamins according to Gamborg B5 medium [32], 0.35 M glucose (POCH), 30 mM sucrose (POCH), 30 mg l^−1^ adenine (Merck), 0.5 MES (Merck), 0.5 mg l^−1^ 2,4-D ( Merck), 0.8 mg l^−1^ NAA (Merck) and 1.0 mg l^−1^ BA (Merck); pH 5.6; filtered (0.22 µm membrane)	protoplast culture	4 °C, dark
Bras5 [33] with modifications	Gamborg B5 macro- and microelements with vitamins [32] (Duchefa Biochemie), 0.3 M glucose (POCH), 0.1 M mannitol (Merck), 0.3 mg l^−1^ 2,4-D (Merck), 1.0 mg l^−1^ NAA (Merck) and 1.0 mg l^−1^ BA (Merck); pH 5.8; filtered (0.22 µm membrane)	protoplast culture	4 °C, dark
sodium alginate solution	0.4 M mannitol (Merck), 2.8% (*w/v*) alginic acid sodium salt (Merck); pH 5.8; filtered (0.22 µm membrane)	protoplast embedding	RT
Ca-agar medium	40 mM calcium chloride (POCH), 0.4 M mannitol (Merck), 1% (*w/v*) agar (Biocorp, Poland); pH 5.8; autoclaved	alginate gelation	RT
sodium citrate solution	20 mM sodium citrate (POCH), 0.2 M mannitol (Merck); pH 5.8; autoclaved	callus microcolonies release	RT
CPPD2	full macro- and microelements, and ¼ of organic acids according to Kao and Michayluk [31] (Duchefa Biochemie), vitamins according to Gamborg B5 medium [32], 0.1 M sucrose (POCH), 0.17 M mannitol (Merck), 250 mg l^−1^ casein enzymatic hydrolysate (Merck), 0.5 mg l^−1^ 2,4-D (Merck), 0.2 mg l^−1^ NAA (Merck) and 0.5 mg l^−1^ BA (Merck), 0.2 mg l^−1^ zeatin^a^ (Duchefa Biochemie); pH 5.6; autoclaved	purification of microcolonies	4 °C, dark
P [24] with modifications	MS micro- and macroelements including vitamins [28] (Duchefa Biochemie), 0.17 M mannitol (Merck), 30 mM sucrose (POCH), 1.0 mg l^−1^ NAA (Merck), 1.0 mg l^−1^ 6-(γ,γ-Dimethylallylamino)purine (2iP; Merck), 0.02 mg l^−1^ gibberellic acid (GA_3_; Merck) and 0.28% (*w/v*) Gelrite (Duchefa Biochemie); pH 5.8; autoclaved	callus culture and shoot regeneration	RT
RBras3	Gamborg B5 micro- and macroelments with vitamins [32] (Duchefa Biochemie), 2.0 mg l^−1^ glycine (Merck), 0.1 M sucrose (POCH), 50 mg l^−1^ casein enzymatic hydrolysate (Merck), 1.0 mg l^−1^ NAA (Merck), 1.0 mg l^−1^ 2iP (Merck), 0.05 mg l^−1^ GA_3_ (Merck) and 0.28% (*w/v*) Gelrite (Duchefa Biochemie); pH 5.8; autoclaved	callus culture and shoot regeneration	RT
RBras4	MS micro- and macroelements including vitamins [28] (Duchefa Biochemie), 0.1 M sucrose (POCH), 0.2 mg l^−1^ indole-3-acetic acid (IAA; Merck), 1.0 mg l^−1^ zeatin^a^ (Duchefa Biochemie) and 0.28% (*w/v*) Gelrite (Duchefa Biochemie); pH 5.7–5.8; autoclaved	callus culture and shoot regeneration	RT

*RT* room temperature

^a^zeatin added after sterilization of medium

### Protoplast isolation and culture

Protoplasts were isolated from leaf blades of 4-week-old in vitro grown plants. Plant material was weighted (g) and then cut into fine pieces and pre-treated in 8 ml of plasmolysis solution (Table 2) for one hour and then, incubated in 8 ml of ESC enzyme solution (Table 2) for 16 h on a gyratory shaker (30 rpm; Rotamax 120, Heidolph Instruments, Germany) at 26 ± 2 °C in the dark. Then the protoplasts were separated from undigested tissues by filtration through a nylon mesh (100 µm; Millipore, USA) and centrifuged (1000 rpm for 5 min; MPW-223e, MPR Med Instruments, Poland; rotor type: MPR no 12,485). The pellet was resuspended in 8 ml of sucrose/MES solution (Table 2) and overlaid with 2 ml of W5 solution (Table 2) for gradient centrifugation (1200 rpm for 10 min). Undamaged protoplasts localized in the interphase between sucrose and W5 solution were transferred into a fresh tube, washed two times by centrifugation (1000 rpm for 5 min each); firstly in 10 ml of W5 solution, and then in 10 ml of CPPO1 culture medium (Table 2). After the purification step, protoplast yield was determined by cell counting, using Fuchs Rosenthal hemocytometer chamber. The working density of protoplasts was adjusted to 8 × 10^5^ protoplasts per ml. Then protoplasts were embedded in calcium alginate layers according to Kiełkowska and Adamus [21]. Equal volumes of protoplast suspension in CPPO1 culture medium and sodium alginate solution (Table 2) were mixed carefully. Alginate layers were obtained by spreading 400 µl protoplast-alginate mixture onto 60 mm Petri dishes containing calcium-agar medium (Table 2). After 1h incubation at room temperature, gelated alginate layers were transferred to 60 mm Petri dishes containing 4 ml of appropriate culture medium (either CPPO1, Bras4 or Bras5 – Table 2). In order to maintain aseptic conditions of the cultures, 200 mg l^−1^ cefotaxime (Polfa Tarchomin SA, Poland) was added to all media. Cultures were incubated in the dark at 24 ± 2 °C. The culture media were renewed once after 10 days of culture.

### Shoot regeneration and plant acclimatization

After 30 days of culture protoplast-derived microcalli were counted (crudes ≥ 0.5mm) on every layer and then were released from alginate matrix by incubation in 8 ml of sodium citrate solution (Table 2) for one hour. The obtained suspension was then centrifuged at 800 rpm for 5 min (MPW-223e, MPR Med Instruments, Poland; rotor type: MPR no 12,485) in order to remove alginate residues and citrate solution. The pellet was then washed in 8 ml of CPPD2 medium (Table 2). Callus derived from one alginate layer was resuspended in 3–4 ml of the CPPD2 medium and plated on filter paper placed in 90 × 15 mm Petri dish with 20 ml of regeneration medium (either P, RBras3 or RBras4 – Table 2). After two weeks the filter paper was removed. Developing shoots were transferred to fresh medium every 3 to 4 weeks. Cultures were maintained at 24 ± 2 °C with a 16h photoperiod at a light intensity of 40 μmol m^−2^ s^−1^. During subsequent passages on to regeneration media, shoots produced roots. Rooted plantlets were planted into multipots filled with moistened coconut substrate (Ceres International Ltd., Pyzdry, PL) and transferred into the climatic chambers SANYO MLR-352H (Sanyo Electric Biomedical Co. Ltd., JP) set up for 19 ± 2 °C with a 16-h photoperiod, a light intensity of 45 μmol m^−2^ s^−1^, and an air humidity of 90%. The plants were acclimatized to *ex vitro* conditions for 2 weeks by a gradual reduction of the air humidity to the final value of 70%.

### Flow cytometry analyses of the regenerants

The ploidy level of the regenerants was estimated using flow cytometry. Briefly, approximately 500 mg of leaf tissue from *in-vitro*-cultured plants was cut with a razor blade in the presence of 1 ml of a lysis buffer (10 mM Tris, 2 mM MgCl_2_·6H_2_O, 50 mM sodium chloride, 0.1% (v/v) TRITON X-100, pH 7.0). The lysis buffer was supplemented with a 1 ml 4′,6-diamidino-2-phenylindole dihydrochloride (DAPI, Merck) solution (10 mg DAPI in 10 ml of water). The suspension was filtered through a nylon filter (pore size 30 μM, Millipore), incubated for 5 min at room temperature, and measured for the relative nuclear DNA content using Partec PA II (Partec GmbH, Münster, Germany). As a reference standard leaves from seed-derived plants of certain *B. oleracea* varieties were used.

### Data collection and statistical analysis

The single experiment consisted of three to fifteen independent protoplast isolations with a single treatment represented by five Petri dishes. Isolation yield was determined using a hemocytometer.

(Heinz Herenz, Germany) and presented as the number of protoplasts per gram of fresh weight (FW). The viability of protoplasts was estimated by staining with fluorescein diacetate (FDA, Merck) approximately 1 h after isolation (day 1) and five days after isolation (day 5). The protocol for protoplast staining was as follows: 15 μl of 0.3% filter-sterilized FDA-acetone stock solution was dissolved in 1 ml of culture medium to prepare FDA working solution. 100 μl of that solution was added to the culture of embedded protoplasts and left for 15 min in the dark. Viability was expressed as a percentage of protoplasts with green fluorescence out of total observed cells. Observations of viability were done on a minimum of 500 cells per treatment. To observe resynthesis of the cell wall, calcofluor white M2R (Merck) was used for cellulose staining. 4 µl of 0.01% water solution of dye was added to the culture dish with protoplasts (filled with 4 ml of culture media) and incubated for 15 min in the dark. Observations were made for *B. oleracea* var. *viridis* after 48 and 72h of culture. Observations under calcofluor were done on a minimum of 500 cells per treatment and were done for cells cultured on CPPO1 and Bras5 media.

Plating efficiency was estimated on 5th (day 5) and 15th day (day 15) of culture and was expressed as a percentage of dividing protoplast-derived cell colonies per total number of observed undivided cells and cell colonies. Observations of plating efficiency were done on minimum 400 objects per treatment. Regeneration frequency was calculated as a percentage of shoots regenerated from callus per total number of calli cultured on the regeneration medium.

All microscopic observations were performed under an inverted Leica DMi8 microscope (Leica Microsystems, Germany) or Carl Zeiss Axiovert S100 microscope (Carl Zeiss, Germany) with a suitable filter set for visualization of fluorescein fluorescence (FITC; λEx = 460–500 nm, λEm = 512–542 nm) and calcofluor white M2R (λEx = 320–360 nm, λEm = 410–450 nm).

Collected data were subjected to an analysis of variance (ANOVA) with separation of means done using Tukey–Kramer post-hoc test. If assumptions of normality and homogeneity of variances were not met, the non-parametric Kruskal–Wallis test followed by the post hoc Dunn’s multiple comparison test were used.

All statistics were calculated with Statistica ver. 13.3 (TIBCO Software Inc., Palo Alto, CA, USA) software at *P* ≤ 0.05. The data are presented as a mean ± standard error (SE).

## Results

### Yield, viability and cell wall re-synthesis of isolated protoplasts

**Fig. 1:**
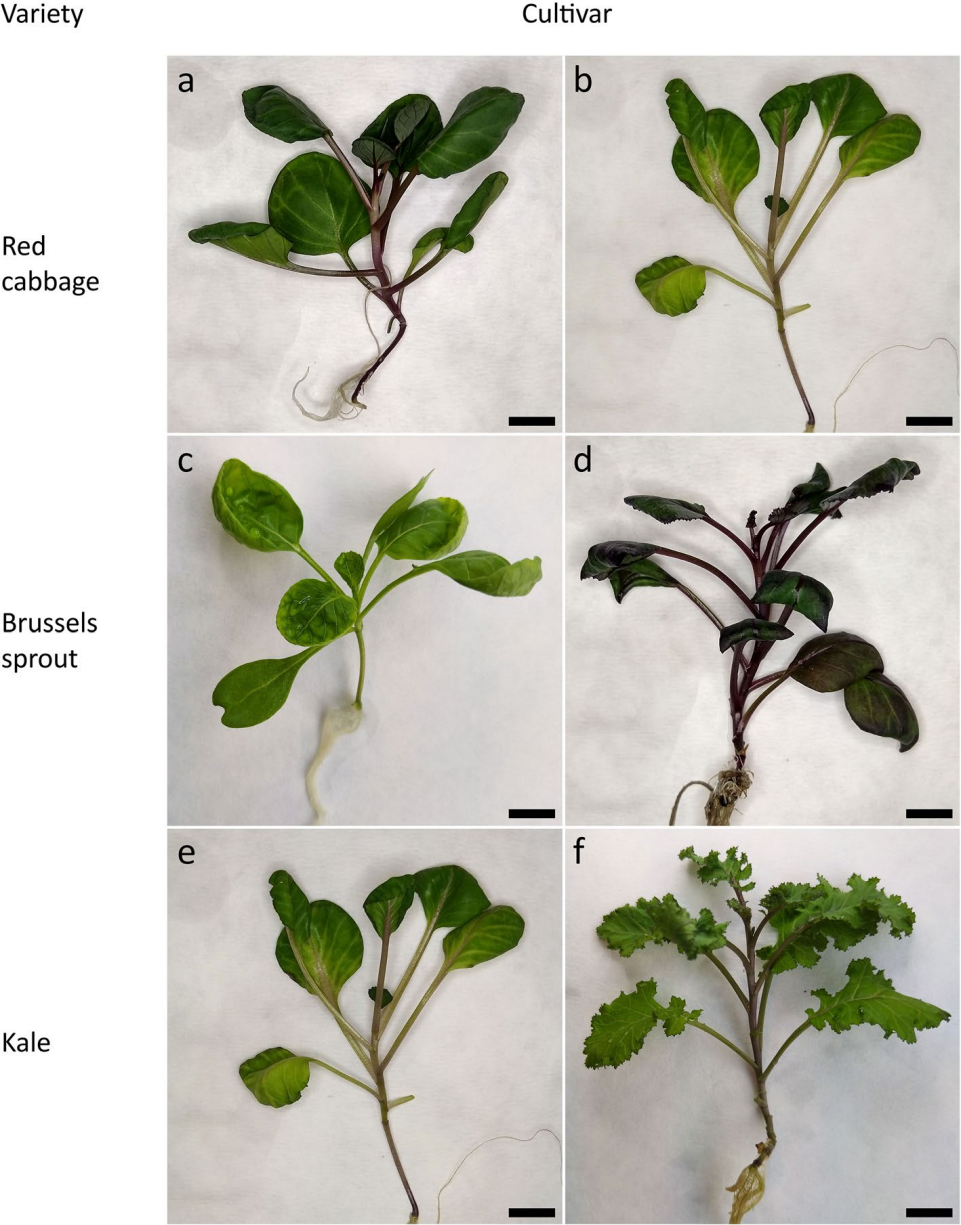
Donor plants for protoplast isolation; red cabbage: ‘Haco’ (**a**), ‘Kalibos’ (**b**); Brussels sprout: ‘Casiopea’ (**c**), ‘Red’ (**d**); kale: ‘Kapral’ (**e**), ‘Scarlet’ (**f**). Scale: 1 cm

**Fig. 2:**
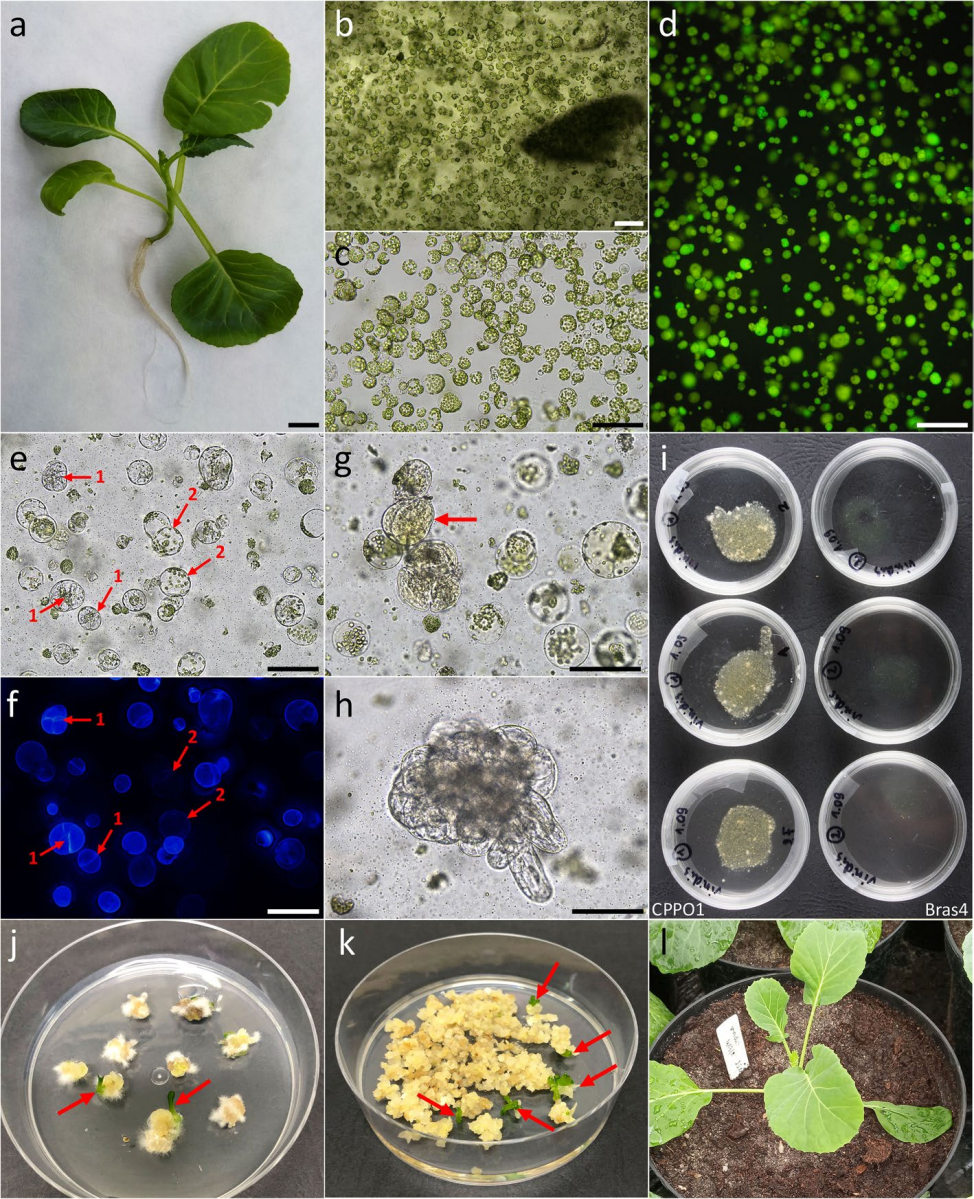
Protoplast cultures of *B. oleracea var. viridis* ‘Vates’: **a** five-week old donor plant; **b**-**c** freshly isolated leaf protoplasts before (**b**) and after (**c**) gradient centrifugation; **d** light-green fluorescence of viable protoplasts stained with FDA; **e** first cell divisions observed after 72 h of culture (**1**) and non-dividing cells (2); **f** cell wall reconstruction tracked by calcofluor white staining – fluorescence shows distribution of cellulose on the surface of protoplasts after 72 h of culture, and points on cytokinesis commencement (**1**) or incomplete cell wall reconstruction (2); **g** cell colony formation on the fifth day of culture (pointed by red arrow); **h** multi-cellular colony on the fifteenth day of culture; **i** protoplast-derived callus with a clearly visible effect of culture media on protoplast culture development; **j**-**k** indirect shoot organogenesis (pointed by red arrows) after 2–3 months on regeneration medium P; **l** protoplast-derived 4-month-old acclimatized plant grown under greenhouse conditions. Scale: **a** 1 cm; **b**-**h** 100 µm

The leaves from in vitro grown plants (Figs. 1a-f and 2a) of all cultivars were an effective source of tissue for protoplast isolation (Fig. 2b-c). The average yield of protoplasts was 2.5 ± 0.3 × 10^6^ cells per g of FW (Table 3). The mean protoplast yield varied considerably between cultivars, and for ‘Haco’ was approximately six-fold higher (5.5 ± 2.2) than for the least efficient cultivar ‘Kapral’ (0.9 ± 0.1).

**Table 3:** Yield of mesophyll-derived protoplasts of seven cultivars of *Brassica oleracea* L

Cultivar	*n*	Protoplast yield (× 10^6^/g FW ± SE)
Haco	3	5.5 ± 2.2 ab
Kalibos	3	2.9 ± 0.6 a-c
Casiopea	12	1.8 ± 0.5 a-c
Red	3	6.1 ± 0.8 a
Kapral	4	0.9 ± 0.1 c
Scarlet	9	3.5 ± 0.4 a
Vates	15	1.4 ± 0.2 bc
Total/Mean	49	2.5 ± 0.3

Means followed by the same letters within a column were not significantly different at *P* ≤ 0.05

*FW* fresh weight, *n* number of independent protoplast isolations

**Fig. 3:**
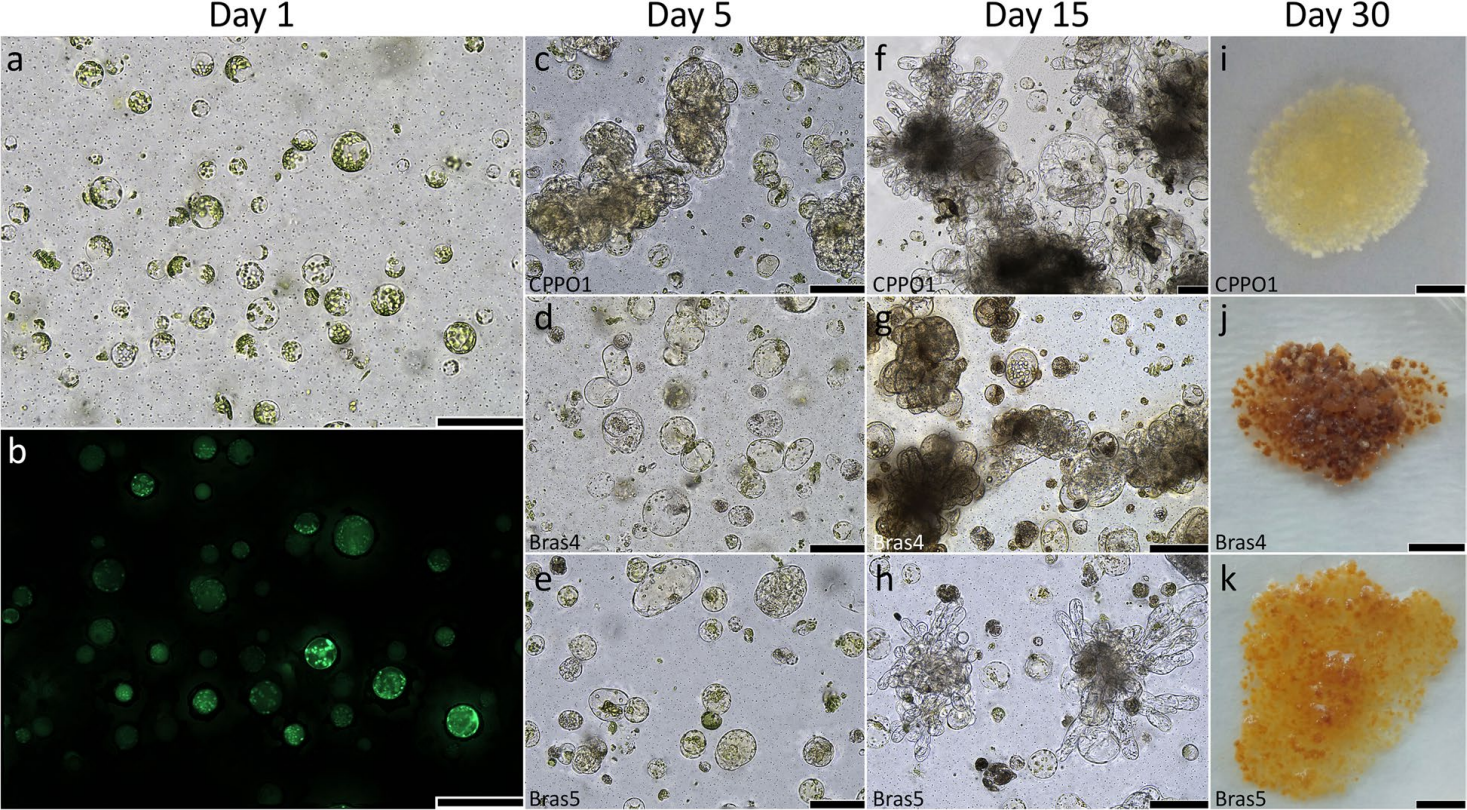
Effect of culture media on protoplast development and protoplast-derived callus formation based on red cabbage ‘Kalibos’. **a**-**b** Alginate embedded protoplasts and their viability in the first day of culture; **c**-**e** cell divisions in five-day-old cultures; **f**–**h** cell colony formation in fifteen –day-old cultures; **i**-**k** protoplasts-derived callus overgrowing alginate layers. Scale: **a**-**h** 100 µm; **i**-**k** 5 mm

The viability of alginate embedded protoplasts, estimated in 1 h-old cultures, was high regardless of cultivar, and varied between 84.6% for ‘Vates’ (Fig. 2d) and 93.6% for ‘Kalibos’ (Table 4, Fig. 3a-b). A decrease of viability was observed after five days of culture for six cultivars, in each of the tested culture medium. The most prominent decrease of viability in relation to the first day of culture was observed for ‘Red’ (from 89.0% to 79.4%), whereas the lowest was noted for ‘Vates’ (from 84.6% to 82.8%). The mean viability of ‘Scarlet’ protoplasts did not change within the first days of culture. No significant differences in protoplast viability were observed for the tested media (Table 4). In general, the highest viability of protoplasts in the five-day old cultures was observed for Bras5 medium (86.8%).

**Table 4:** Effect of cultivar and culture medium on protoplast viability in *Brassica oleracea* L

Factor	Protoplast viability (% ± SE)		
	***n***	**in 1-day-old cultures**	**in 5-day-old cultures**
**Cultivar**			
Haco	9	88.8 ± 1.7 a-c	79.7 ± 5.3 bc
Kalibos	9	93.6 ± 0.7 a	87.7 ± 2.0 a-c
Casiopea	12	90.4 ± 0.5 ab	88.2 ± 1.3 ab
Red	9	89.0 ± 2.7 ab	79.4 ± 4.7 bc
Kapral	16	87.1 ± 0.4 bc	83.7 ± 1.0 bc
Scarlet	16	90.6 ± 0.9 ab	90.6 ± 0.6 a
Vates	31	84.6 ± 0.7 c	82.8 ± 0.7 c
**Culture medium**^**a**^			
CPPO1	44	88.3 ± 0.7	83.9 ± 1.1
Bras4	31	88.0 ± 0.8	83.8 ± 1.5
Bras5	27	88.2 ± 1.1	86.8 ± 1.7
Total/Mean	102	88.2 ± 0.5	84.7 ± 0.8

Means followed by the same letters within a column were not significantly different at *P* ≤ 0.05

*N* number of independent protoplast isolations

^a^ The means represent averages of seven cultivars

**Fig. 4:**
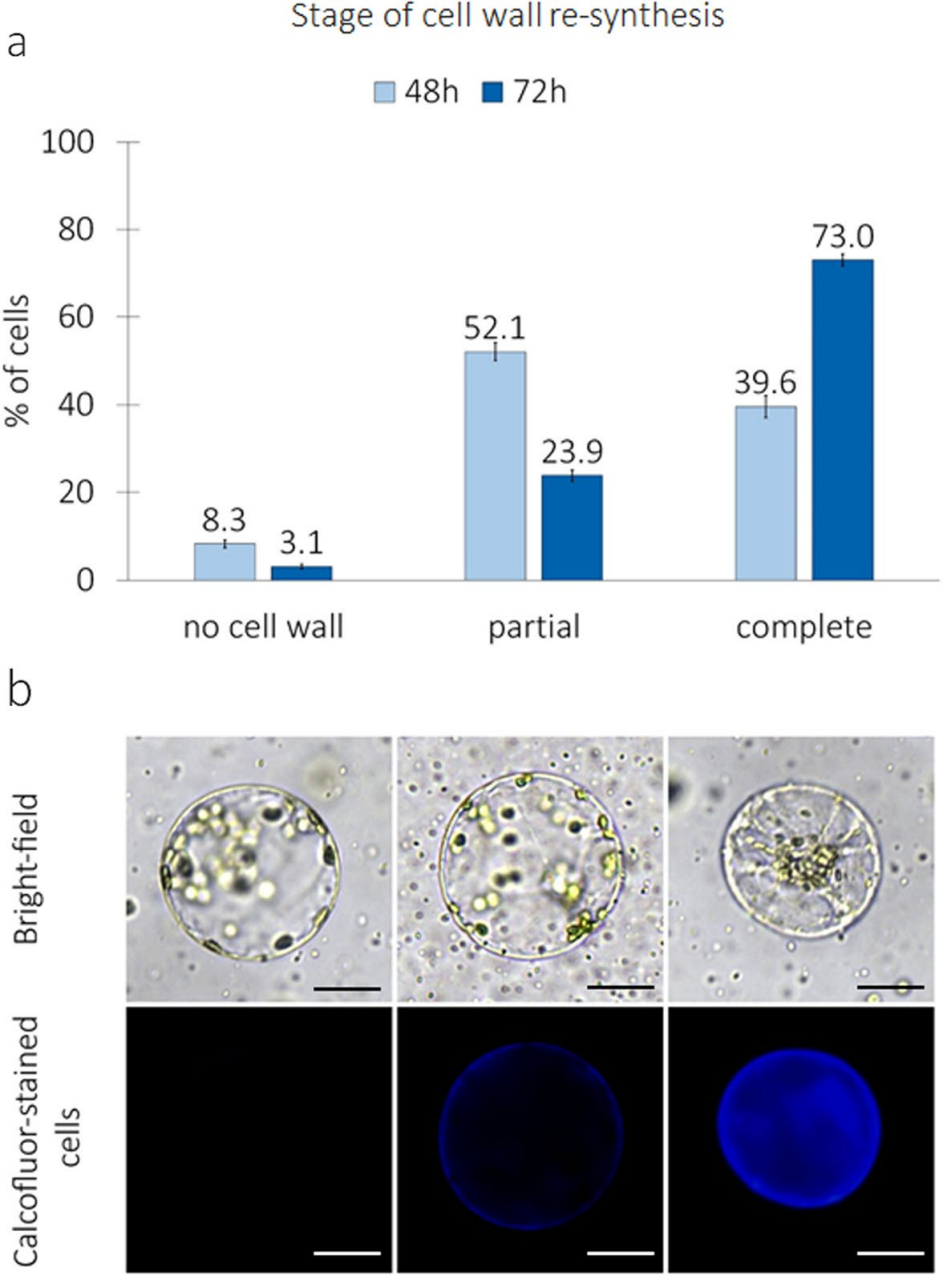
Cell wall re-synthesis in protoplast cultures of *B. oleracea* var. *viridis* ‘Vates’. **a** the percentage of cells with no cell wall re-synthesis, partial cell wall re-synthesis and complete cell wall re-synthesis observed in 48th and 72nd hour of culture. Bars represent means (from CPPO1 and Bras5 media jointly) ± SE obtained from at least three independent experiments; **b** stages of cell wall re-synthesis tracked by calcofluor white staining – blue fluorescence shows distribution of cellulose on the surface of cells. Stages arranged in order of presentation on a chart. Scale: 25 µm

Regardless of the culture medium used, collard protoplasts showed the ability to completely re-synthesize the cell wall (Fig. 4a). The blue fluorescence of cellulose after calcofluor treatment was the evidence for the cell wall re-synthesis process (Fig. 4b). While cells devoid of a cell wall were identified at both time points, the majority of protoplasts commenced the process of cell wall re-synthesis. In 48-h-old cultures, cells with a complete and partially reconstituted wall represented 91.7% of the observed cells. After 72 h of culture, cells with a complete and partial re-synthesis represented 96.9% of observed cells (73.0 and 23.9%, respectively).

### Effect of cultivar and culture medium on plating efficiency and microcallus formation

Seven cultivars were compared to evaluate the plating efficiency in *B. oleracea* protoplast cultures in three culture media. Change in protoplast shape was observed around third and fourth day of culture. First mitotic divisions occurred in the fourth day of culture for all cultivars (Fig. 2e-f) and differences in plating efficiency between tested cultivars were observed. On the 5th day the average plating efficiency was 46 ± 3.0% (Table 5, Additional file 1: Tab. S1 and Tab. S2, Fig. 3c-e) and increased gradually with the duration of culture, reaching an average of 62.1 ± 2.0% on the 15th day (Fig. 3f-h). In five-day-old cultures, the highest plating efficiency was scored for ‘Kapral’ (79.6 ± 1.2%) and ‘Vates’ (66.3 ± 5.0%) while for other cultivars it ranged between 21–29%. In fifteen-day-old cultures plating efficiency was more evenly distributed and ranged from 45 to 78% (Table 5). At both time points there were no differences in plating efficiency, regardless of culture medium and cultivar used (Table 5, Additional file 1: Tab. S1).

**Table 5:** Effect of cultivar and culture medium on plating efficiency in protoplast cultures of *Brassica oleracea* L

Factor	Plating efficiency (% ± SE)		
	***n***	**in 5-day-old cultures**	**in 15-day-old cultures**
**Cultivar**			
Haco	9	29.3 ± 3.6	45.1 ± 4.3 ^1, 4^
Kalibos	9	26.7 ± 3.9 ^1, 5^	56.8 ± 6.6
Casiopea	12	28.6 ± 7.2 ^2, 6^	49.6 ± 3.9 ^2, 5^
Red	9	27.1 ± 2.2 ^3^	53.7 ± 6.1
Kapral	15	79.6 ± 1.2 ^1, 2, 3, 4^	78.0 ± 1.4 ^1, 2, 3^
Scarlet	16	21.4 ± 3.7 ^4, 7^	53.9 ± 5.0 ^3, 6^
Vates	29	66.3 ± 5.0 ^5, 6, 7^	73.0 ± 3.3 ^4, 5, 6^
**Culture medium**^**a**^			
CPPO1	41	45.4 ± 4.2	64.8 ± 2.5
Bras4	31	47.0 ± 5.6	59.5 ± 4.1
Bras5	27	45.7 ± 6.2	60.8 ± 4.1
Total/Mean	99	46.0 ± 3.0	62.1 ± 2.0

^a^ The means represent averages of seven cultivars, *n*—number of independent protoplast isolations

Number in superscript shared by two cultivars indicate a significant (*P* ≤ 0.05) difference in plating efficiency revealed by Dunn’s multiple comparison test (see Additional file 1: Tab. S2)

Continuous mitotic divisions of the protoplast-derived cells lead to the formation of cell colonies (Fig. 2g-h). Cell colonies developed into microcallus visible to the naked eye (≥ 0.5 mm) at around the fourth week of culture (Fig. 2i). The mean number of microcalli produced per single dish was cultivar- and medium-dependent (Table 6, Additional file 1: Tab. S3 and Tab. S4). Cultivar ‘Red’ was characterized by the highest number of microcalli (60.5 ± 2.2 per dish), whereas the least prolific ‘Kapral’ produced 29.8 ± 0.8 microcalli per dish. The average number of microcalli produced in culture media Bras4 and Bras5 was 9.5% and 4.2% lower than in CPPO1, respectively (Table 6).

**Table 6:** Effect of cultivar and culture medium on microcallus formation in protoplast cultures of *Brassica oleracea* L

Factor	*n*	Number of microcalli formed per alginate layer
**Cultivar**		
Haco	87	42.7 ± 1.8 ^1, 2, 3^
Kalibos	79	51.9 ± 2.9 ^4, 5^
Casiopea	156	42.4 ± 0.9 ^6, 7, 8, 9^
Red	82	60.5 ± 2.2 ^1, 6, 10, 11^
Kapral	56	29.8 ± 0.8 ^2, 4, 7, 10, 12^
Scarlet	133	48.5 ± 1.1 ^8, 12, 13^
Vates	198	31.6 ± 1.4 ^3, 5, 9, 11, 13^
**Culture medium**^**a**^		
CPPO1	359	44.3 ± 1.0 ^1^
Bras4	209	40.1 ± 1.2 ^1^
Bras5	223	42.4 ± 1.4
Total/Mean	791	42.7 ± 0.7

^a^ The means represent averages of seven cultivars

Different letters in superscript shared by two cultivars/media indicate a significant (*P* ≤ 0.05) difference in microcallus formation revealed by Dunn’s multiple comparison test (see Additional file 1: Tab. S4)

*n* number of independent cultures (Petri dish)

### Plant regeneration from protoplast cultures of *Brassica oleracea* L

**Fig. 5:**
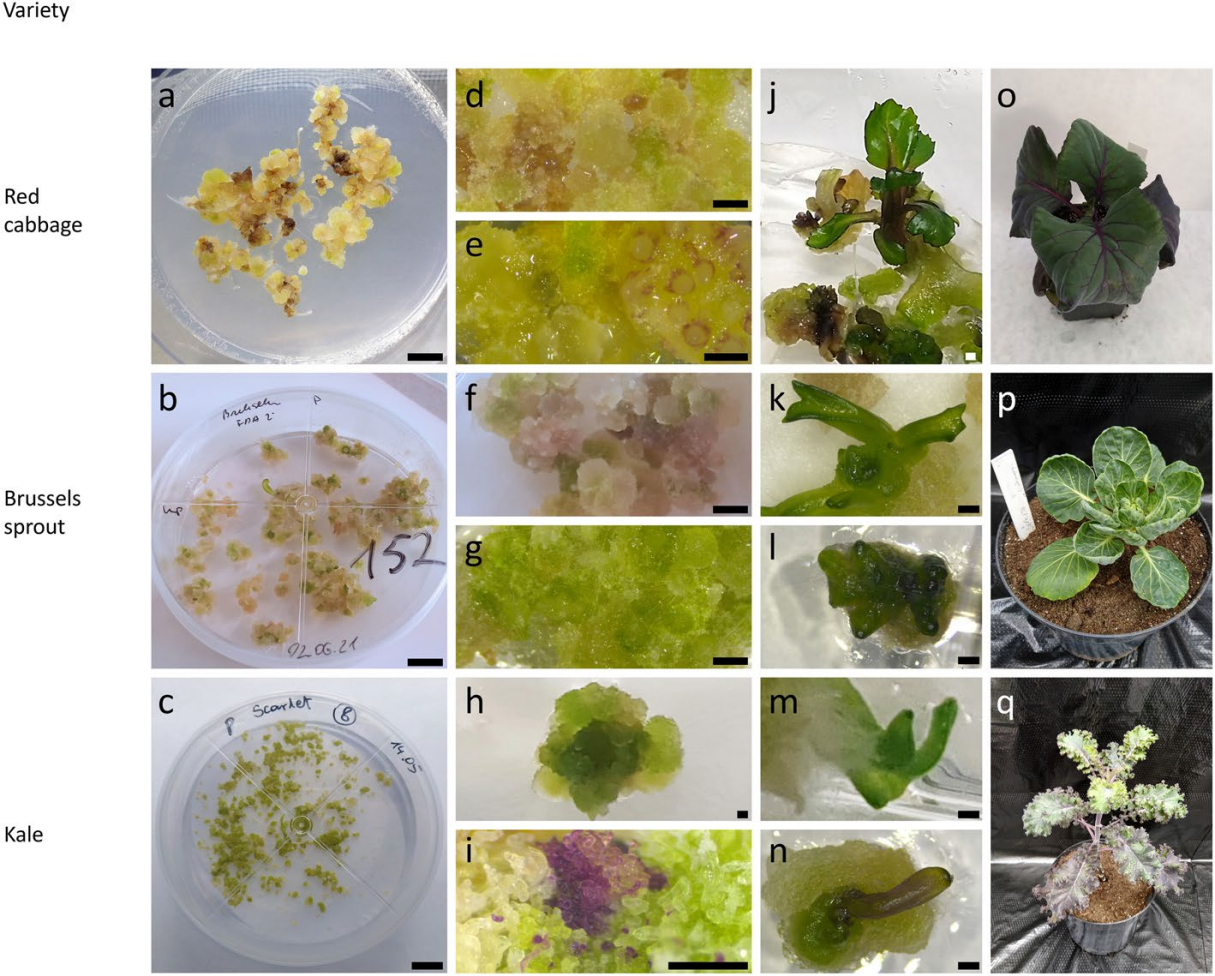
Plant regeneration of protoplast-derived callus and their acclimatization to *ex vitro* conditions: **a**-**n** protoplast-derived callus development and organogenesis: **a** callus of red cabbage ‘Kalibos’ after six weeks on regeneration medium P; **b** eight-week-old callus of Brussels sprout ‘Casiopea’ on regeneration medium P; **c** callus of kale ‘Scarlet’ after four weeks on regeneration medium P; **d**-**e** callus of red cabbage ‘Kalibos’ (**d**) ‘Haco’ (**e**) showing regions with anthocyanin rich cells; **f**-**g** callus of Brussels sprout ‘Casiopea’ (**f**) and ‘Red’ (**g**); **h**-**i** callus of kale ‘Kapral’ (**h**) and ‘Scarlet’ (**i**) with visible anthocyanin rich cell clusters; **j**-**n** indirect shoot organogenesis of red cabbage ‘Kalibos’ (**j**), Brussels sprout ‘Cassiopea’ (**k**) and ‘Red’ (**l**), kale ‘Kapral’ (**m**) and ‘Scarlet’ (**n**); **o**-**q** four-month-old acclimatized plants: red cabbage ‘Kalibos’, Brussels sprout ‘Casiopea’ and kale ‘Scarlet’, respectively. Scale: **a**-**c** 1 cm; **d**-**n** 1 mm

Calli (Figs. 2i, and 3i-k) obtained in each treatment were released from alginate layers and placed on three solid regeneration media P, RBras3 and RBras4. Upon reaching four weeks of culture on the regeneration medium, many callus clumps expanded in size and turned green (Fig. 5a-c) with an occasional formation of anthocyanin rich cell clusters. However, some callus clumps changed to a brown color and did not develop further (Fig. 5d-e). The plant regeneration efficiency was cultivar- and medium-dependent (Table 7, Additional file 1: Tab. S5 and Tab. S6). In six of tested cultivars the growth of green callus was sustained, leading to the development of morphogenic structures (Fig. 5f-i), followed by the regeneration of shoots (Fig. 5j-n). The callus of ‘Haco’ failed to regenerate on every of the tested regeneration media. Among cultivars displaying regenerative ability, ‘Kalibos’ was characterized by the highest rate of shoot formation (6.6 ± 1.9%). The frequency of shoot development for ‘Red’ was the lowest and did not exceed 0.1%. The average shoot regeneration frequency (SRF) observed for other cultivars was more uniform and ranged from 1.6% for ‘Casiopea’ to 3.5% for ‘Kapral’. The mean efficiency of regeneration on RBras3 and RBras4 was lower when compared to P medium, whereas the influence of protoplast culture medium used on the plant regeneration was not apparent (Table 7).

**Table 7:** Shoot regeneration from the protoplast-derived callus of seven cultivars of *Brassica oleracea* L

Factor	*n*	Shoot regeneration (% ± SE)
**Cultivar**		
Haco	85	0.0
Kalibos	81	6.6 ± 1.9
Casiopea	106	1.6 ± 0.7
Red	84	0.1 ± 0.0
Kapral	59	3.5 ± 1.1
Scarlet	72	2.3 ± 0.8
Vates	107	3.3 ± 1.0
**Protoplast culture medium**^**a**^		
CPPO1	187	3.7 ± 0.9
Bras4	166	2.4 ± 0.6
Bras5	156	2.3 ± 0.7
**Regeneration medium**^**a**^		
P	186	6.4 ± 1.0 ^1, 2^
RBras3	144	0.9 ± 0.6 ^1^
RBras4	179	0.7 ± 0.2 ^2^

^a^ The means represent averages of six cultivars

Number in superscript shared by two cultivars/media indicate a significant (*P* ≤ 0.05) difference in shoot formation revealed by Dunn’s multiple comparison test (see Additional file 1: Tab. S6)

*N* number of independent cultures (Petri dish)

A more detailed analysis of regeneration capacity in dependency from protoplast culture medium and regeneration medium showed differences in response of particular cultivars (Additional file 1: Tab. S7). ‘Vates’ showed the most efficient shoot regeneration, 19.7%, in CPPO1 medium-derived callus cultures maintained on the P regeneration medium (Fig. 2j-k), whereas RBras4 medium-derived callus cultures maintained on P medium were characterized by much lower SRF (1.8%). Similarly, for ‘Kapral’ more than two-fold higher SRF was observed in combination CPPO1 culture medium + P regeneration medium when compared to RBras4 + P (11.1% vs. 5.1%). SRF of ‘Casiopea’ was also influenced by the combination of used protoplast culture and regeneration media. The highest number of regenerated shoots was observed in Bras4 medium-derived callus cultures maintained on the P medium (10.9%). A three to five-fold decrease in SRF was noted for both Bras5 + P and CPPO1 + P combination (3.6 and 2.2%, respectively). Moreover, SRF of ‘Casiopea’ on RBras3 was very low (0.3%) and no regeneration on RBras4 was observed. Shoot regeneration of cv. ‘Scarlet’ was limited to P medium, with the highest SRF observed in Bras5 + P medium (9.9%). This media combination proved to be also the most suitable for shoot regeneration of ‘Kalibos’ (16.5%; Additional file 1: Tab. S7). Obtained shoots during subsequent passages produced roots, and only these were subjected to the acclimatization. Successfully acclimatized regenerants were subjected to ploidy analyses. In total, 176 regenerants (Figs. 2l, and 5o-q) representing five cultivars were subjected to flow cytometry analysis (Table 8). The majority of regenerants were diploid (79.5%), however, tetraploids were also identified (18.2%). Moreover, 2.3% of regenerants were characterized by mixed ploidy (2x − 4x).

**Table 8:** Ploidy status of protoplast-derived shoots of five *Brassica oleracea* L. cultivars

Cultivar	Regeneration medium	Number of analyzed plants	Ploidy (number of analyzed samples)
Kalibos	*P*	26	2x (9), 4x (16), 2x − 4x (1)
	RBras4	2	2x (1), 4x (1)
Casiopea	*P*	33	2x (31), 4x (1), 2x − 4x (1)
Kapral	*P*	28	2x (16), 4x (12)
	RBras4	1	2x
Scarlet	*P*	49	2x (47), 4x (1), 2x − 4x (1)
Vates	*P*	20	2x (19), 4x (1)
	RBras3	3	2x
	RBras4	14	2x (13), 2x − 4x (1)
Total (%)		176	2x (79.5%), 4x (18.2), 2x-4x (2.3%)

## Discussion

### Yield and viability of isolated protoplasts

The present study has developed a successful protocol for plant regeneration via indirect organogenesis from leaf protoplasts in six of the seven tested cultivars of *Brassica oleracea*. The use of leaf mesophyll as a source of protoplasts has been previously reported in *Brassica* species, including *B. oleracea* [22, 24–26], *B. napus* [24] and occasionally *B. rapa* [34]. Hussain et al. [26] determined that true leaves are a more efficient source of protoplasts compared to cotyledons in their study of five *B. oleracea* varieties. Our research involved slight adjustments to the enzyme solution proposed by Kiełkowska and Adamus [35], such as higher concentrations of CaCl_2_ and MES.

These adjustments resulted in a very high average yield of 2.5 × 10^6^ cells per gram of fresh weight. Consistent with previous findings [18, 36], the protoplast yield was influenced by the genotype, aligning with existing studies on protoplast isolation from mesophyll of *B. oleracea* [16, 21, 26]. Moreover, true leaves proved to be a suitable source of tissue for protoplast isolation from collard, a much less extensively studied variety of *B. oleracea*. The growth and development of protoplast cultures is greatly influenced by the density of viable protoplasts. A too low or too high density may inhibit cell divisions, and therefore, callus formation due to an unbalanced release of growth factors [18]. A density of 2 to 5 × 10^5^ protoplasts/ml is considered the most optimal for *Brassicaceae* species [16, 21, 22, 35, 37, 38].

Even with the lowest yield of 0.9 × 10^6^ cells/g FW from the cultivar 'Kapral', it was still sufficient for establishing protoplast culture at the optimal density. Hence, the optimized protocol for tissue preparation, enzymatic digestion, and protoplast purification can be considered suitable for various *B*. *oleracea* varieties. The isolated and purified protoplasts from all seven cultivars displayed high viability, both on the day of isolation and after five days in culture.

The method used for protoplast culture can significantly impact cell divisions and the formation of callus. Protoplasts cultured in liquid medium are prone to aggregation leading to an overproduction of toxic metabolites and formation of non-homogeneous callus [39, 40]. To address this issue, embedding protoplasts in a semi-solid medium such as agar [41], agarose [42], or calcium alginate [43] has been widely practiced to provide a physical separation of cells in protoplast cultures across various plant species [44–48]. While all three protoplast embedding systems have been employed in *Brassica* species [24, 26, 27], the use of alginate has consistently enhanced cell division and plating efficiency, particularly in *B. oleracea* [16, 21–23]. To ensure both high protoplast viability and cell divisions, we chose low-viscosity and filter-sterilized alginate as a solidifying agent. Previous studies by Kiełkowska and Adamus [49] have emphasized the significant improvement in plating efficiency with this embedding method in three *B. oleracea* var. *capitata* cultivars.

### Effect of cultivar and culture medium on cell division and microcallus formation

The composition of the culture medium is the key factor ensuring viability of protoplasts and high plating efficiency. The appropriate concentration of micro-, macronutrients and vitamins, together with a suitable osmotic stabilizer, is crucial for viability and vigor of protoplasts in the first stage of culture, when cell wall resynthesis occurs [18, 50]. Our research found that all culture media tested in this study, i.e. CPPO1, Bras4 and Bras5, did not negatively impact survival ratio of protoplasts on the 5th day of culture. It suggests that media compositions, particularly the osmotic pressure provided by a concentration of 0.3 to 0.4M glucose and mannitol/sucrose, were well-suited for the initial stage of protoplast culture of *B. oleracea* cultivars.

Cell wall reconstruction is one of the first stages of protoplast development, allowing further mitotic divisions and differentiation. The process of cellulose reconstruction in cabbage protoplasts is cultivar specific and non-synchronous. At the same time, cells with incomplete cell wall reconstruction can be observed during culture, as well as cells with completely resynthesized cellulose over the entire cell surface. Previous studies have shown that in cabbage, almost 80–90% of the cells have rebuilt the cell wall by the seventieth hour of culture [22], which was also confirmed by our observations of *B. oleracea* var. *viridis* culture in each tested culture medium.

Protoplasts from all seven cultivars underwent first divisions within the first 5 days in all tested culture media. Moreover, the plating efficiency increased over time, reaching a mean of 46% on the 5th day and 62% on the 15th day of culture. These results are in line with plating efficiencies reported by Pauk et al. [27] for *B. campestris* and *B. napus,* as well as by Glimelius [51] for *B. oleracea*, at a similar culture stage. Interestingly, many studies reported much lower plating efficiencies in *B. oleracea*, ranging from 3.7% to 30% on the 5th-7th day of culture [21, 25, 37, 52], and from 10.9% to 33% on the 15th day [21, 49, 52]. Our observations, consistent with previous studies on *Brassica* [21, 23, 26, 49], highlight the significant role of genotype on protoplast response to culture conditions. Specifically, protoplasts of two cultivars, i.e. ‘Kapral’ and ‘Vates’, underwent divisions more frequently and much faster compared to other cultivars. Surprisingly, the frequent cell divisions of these cultivars did not enhance the efficiency of microcallus formation. This effect could be attributed to a robust production of reactive oxygen species and/or phenolics during extensive divisions in the initial stages of protoplast cultures [53]. Consequently, this could result in the oxidative stress-related inhibition of protoplast growth and divisions at later stages of culture [54].

The composition and concentration of plant growth regulators (PGRs) and additional supplements in the culture medium is known to be an important factor in promotion of cell divisions and microcallus formation [18, 39]. In all of the tested culture media, 2,4-D and NAA were used as a source of auxins, as often recommended and applied in *B. oleracea* [16, 21, 23, 51, 55]. Similarly to study of Dietert et al. [55] we observed that higher concentrations of PGRs, 2,4-D in particular, may have a slightly detrimental effect on microcallus proliferation in the cultivars under investigation.

### Plant regeneration from protoplast cultures

The remarkable diversity of *B. oleracea* has been widely acknowledged, particularly regarding the success rate of in vitro regeneration from different genotypes and explants within this species. Regeneration of shoots from protoplast cultures of cabbage is challenging due to the dominant influence of genotype on plant regeneration protocols, presenting a persistent issue [23, 49, 56]. To date, several studies have shown successful shoot regeneration in various *Brassica* species, although shoot formation efficiency can vary significantly, even within the same species. For example, Stajič et al. [23] reported relatively high shoot formation efficiency of 23.5%, but only in one of five studied *B. oleracea* cultivars; the remaining cultivars did not regenerate. Similarly, in the study of Kiełkowska and Adamus [49], the shoot formation efficiency ranged from 11.4% to 41.6%, with four out of ten accessions failing to produce shoots. The dominant influence of genotype extends beyond *B. oleracea* as demonstrated by Pauk et al. [27]. It has been reported that ten out of thirteen assessed *B. napus* cultivars successfully formed shoots on the regeneration medium. In our study, six of seven *B. oleracea* cultivars produced shoots on regeneration media, though the efficiency of shoot formation was strongly influenced by both: the cultivar, and the regeneration medium, and varied between 0.1% and 6.6%. We did not observe shoot induction of red cabbage ‘Haco’. Similar to previous research on shoot regeneration in *Brassica* [22], the regeneration capacity could generally be associated with a well-structured, dense green callus, while white, loose and browning callus did not exhibit regeneration potential. Interestingly, ‘Haco’ produced a dense green, potentially able to regenerate callus. The composition of the regeneration medium, another key factor to a successful plant regeneration, did not affect the regeneration capacity of ‘Haco’ callus, highlighting the dominant role of genotype on the protoplast-to-plant regeneration capacity.

Typically, a higher cytokinin to auxin ratio (C/A) is necessary for shoot induction, although this requirement can vary depending on the species and/or genotype [57, 58]. Our findings contradict this notion, as we found that medium P, with a C/A ratio of 1:1 had significantly, nearly ten-fold, higher shoot formation efficiency compared to medium RBras4, which had a C/A ratio of 5:1. This aligns with observations of Hussain et al. [26] that high C/A ratios were less effective in inducing organogenesis in *B. oleracea*.

**Fig.6:**
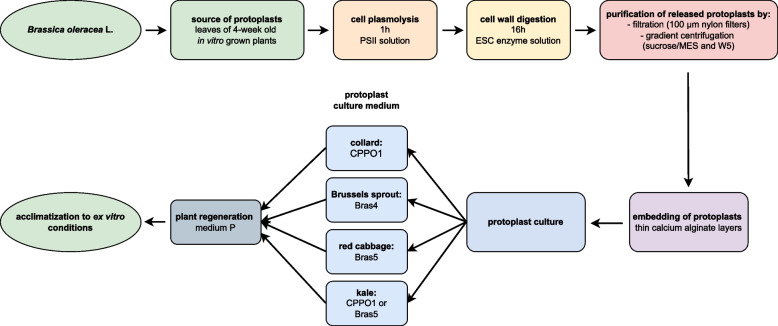
Flow chart showing subsequent steps of the protocol for protoplast-to-plant regeneration of the tested *B. oleracea* varietes

While auxins and cytokinins are widely recognized as the primary PGRs for plant regeneration, the role of other PGR’s in cell differentiation and development should not be underestimated. Generally, gibberellins are not crucial for in vitro plant cultures and are usually not incorporated into the regeneration media due to their potential to hinder the formation of shoots and roots [59]. However, our finding demonstrates that the addition of GA_3_ in low concentrations, not exceeding 0.02 mg l^−1^, can actually promote shoot formation in *B. oleracea*. This observation aligns with several previous research emphasizing the role of GA_3_ in regeneration and transformation protocols of different species, also within *Brassicaceae* family [24, 60, 61]. The choice of exogenously supplied carbon source significantly impacts in vitro plant regeneration processes. Various studies have shown that different carbon sources such as glucose, sucrose and sorbitol play crucial roles in callus induction and regeneration efficiency in various plant species, including cotton, banana and rice [62–65]. The majority of plants rely on sucrose as their main carbon source since it is the predominant carbohydrate produced and moved through the phloem [66, 67]. Indeed, the majority of reported protocols of plant regeneration from protoplasts of *B. oleracea* use 1–3% of sucrose in regeneration media [22–24, 26, 35, 49], rarely glucose [51], but never mannitol. Our results show that the regeneration medium supplemented with reduced sucrose (1%) coupled with mannitol (2%) stimulates more effective shoot regeneration of the studied cultivars of *B. oleracea* compared to the other two tested media supplemented with 3% sucrose. This contradicts the common belief that mannitol’s limited effectiveness, compared to other sugars, is attributed to its osmotic properties and lack of physiological activity, which hinder its ability to support developmental processes as an energy source [68, 69]. Figure 6 presents subsequent steps of the protocol for protoplast-to-plant regeneration of the tested *B. oleracea* varieties i.e. collard, Brussel Sprout, red cabbage and kale.

Flow cytometry analysis of regenerated plants revealed that the majority (79.5%) were diploid; however, tetraploids and mixoploids were also identified. The type of regeneration medium used did not influence polyploidization. The increase in ploidy levels was more likely due to spontaneous fusion after isolation which explains occurrence of tetraploids, however other genetic factors will be responsible for the occurrence of mixoploidy. In vitro-induced disruption of cytokinesis and subsequent fusion of daughter nuclei could cause mixoploidy and genome doubling during callus proliferation, leading to genetically unstable callus cultures. Hence the mixed ploidy of shoots indirectly regenerated from mitotically unstable callus.

## Conclusions

In this study, we successfully obtained high yields of viable protoplasts from seven different *B. oleracea* cultivars representing red cabbage, Brussels sprout, kale, and collard. This was achieved by the use of optimized enzyme solution and culture media. Microcallus was formed from protoplasts of all cultivars, although the quantity of microcalli obtained was dependent on the genotype and culture medium used. Plant regeneration was achieved in six cultivars, while microcallus of red cabbage ‘Haco’ failed to produce shoots. Furthermore, this study presents, for the first time, the complete protocol for protoplast-to-plant regeneration of collard, a very valuable, however, less commonly cultivated variety of *B. oleracea*. With the interest in utilizing collard as a source of recombinant antigens [70], protoplasts could serve as reliable source of explants for both stable and transient transformation, facilitating the production of pharmaceutical proteins, such as B5 recombinant vaccine candidate against smallpox. The newly established procedure sets the stage for more widespread utilization of *B. oleracea* protoplasts and further improvement of this significant crop.

## Supplementary Information

Supplementary Material 1.

## Data Availability

The datasets supporting the conclusions of this article are included within the article or are available from the corresponding author on reasonable request.

## References

[1] Witzel K, Kurina AB, Artemyeva AM. Opening the treasure chest: the current status of research on *Brassica oleracea* and *B. rapa* vegetables from *ex situ* germplasm collections. Front Plant Sci. 2021;12:643047. 34093606 10.3389/fpls.2021.643047 PMC8173032

[2] Farnham MW. Genetic variation among and within United States collard cultivars and landraces as determined by Randomly Amplified Polymorphic DNA markers. J Am Soc Hortic Sci. 1996;121:374–9.

[3] Song KM, Osborn TC, Williams PH. *Brassica* taxonomy based on nuclear restriction fragment length polymorphisms (RFLPs): 2. Preliminary analysis of subspecies within *B. rapa* (syn. *campestris*) and *B. oleracea*. Theor Appl Genet. 1988;76:593–600. 24232282 10.1007/BF00260914

[4] Farnham MW, Davis EH, Morgan JT, Smith JP. Neglected landraces of collard (*Brassica oleracea* L. var. *viridis*) from the Carolinas (USA). Genet Resour Crop Evol. 2008;55:797–801.

[5] Farnham MW, Keinath AP, Smith JP. Characterization of fusarium yellows resistance in collard. Plant Dis. 2001;85:890–4. 30823058 10.1094/PDIS.2001.85.8.890

[6] Farnham MW, Ruttencutter G, Smith JP, Keinath AP. Hybridizing collard and cabbage may provide a means to develop collard cultivars. HortScience. 2005;40:1686–9.

[7] Lian YJ, Lin GZ, Zhao XM, Lim HT. Production and genetic characterization of somatic hybrids between leaf mustard (*Brassica juncea*) and broccoli (*Brassica oleracea*). Vitro Cell Dev Biol - Plant. 2011;47:289–96.

[8] El-Esawi MA. Somatic hybridization and microspore culture in *Brassica* improvement. In: Anis M, Ahmad N, editorss. Plant tissue culture: propagation, conservation and crop improvement. Singapore: Springer; 2016. p. 599–609.

[9] Kumari P, Singh KP, Kumar S, Yadava DK. Development of a yellow-seeded stable allohexaploid *Brassica* through inter-generic somatic hybridization with a high degree of fertility and resistance to *Sclerotinia sclerotiorum*. Front Plant Sci. 2020;11:575591. 33329636 10.3389/fpls.2020.575591 PMC7732669

[10] Shinke T, Yamazaki A, Kudo H, Hosokawa M. Genetic diversification of allohexaploid *Brassica* hybrids (AABBCC) using fertile octoploid with excessive C genome set (AABBCCCC). Planta. 2024;260:71. 39136783 10.1007/s00425-024-04497-w

[11] Jourdan PS, Earle ED, Mutschler MA. Synthesis of male sterile, triazine-resistant *Brassica napus* by somatic hybridization between cytoplasmic male sterile *B. oleracea* and atrazine-resistant *B. campestris*. Theor Appl Genet. 1989;78:445–55. 24227255 10.1007/BF00265310

[12] Zhao Y, Yang D, Liu Y, Han F, Li Z. A highly efficient genetic transformation system for broccoli and subcellular localization. Front Plant Sci. 2023;14:1091588. 36937998 10.3389/fpls.2023.1091588 PMC10018207

[13] Liu Y, Zhang L, Li C, Yang Y, Duan Y, Yang Y, et al. Establishment of *Agrobacterium*-mediated genetic transformation and application of CRISPR/Cas9 genome-editing system to *Brassica rapa* var *rapa.* Plant Methods, 2022;18(1):98. 10.1186/s13007-022-00931-w PMC9356411 35933391

[14] Sheng X, Yu H, Wang J, Shen Y, Gu H. Establishment of a stable, effective and universal genetic transformation technique in the diverse species of *Brassica oleracea*. Front Plant Sci. 2022;13:1021669. 36311069 10.3389/fpls.2022.1021669 PMC9597678

[15] Wu H, Wang J, Zhang K, Zhao Y, Duan Y, Li J, et al. *Brassica napus* BBM-GR transformants from bulk-way transformation. Life Sci Technol. 2023;2:47.

[16] Stajič E, Kiełkowska A, Murovec J, Bohanec B. Deep sequencing analysis of CRISPR/Cas9 induced mutations by two delivery methods in target model genes and the CENH3 region of red cabbage (*Brassica oleracea* var. *capitata* f. *rubra*). Plant Cell Tissue Organ Cult. 2019;139:227–35.

[17] Yu X, Yu J, Lu Y, Li W, Huo G, Zhang J, et al. An efficient and universal protoplast-based transient gene expression system for genome editing in *Brassica* crops. Hortic Plant J. 2024;10:983–94.

[18] Reed KM, Bargmann BO. Protoplast regeneration and its use in new plant breeding technologies. Front Genome Ed. 2021;3:20. 10.3389/fgeed.2021.734951 PMC8525371 34713266

[19] Zhao K-N, Bittisnich DJ, Halloran GM, Whitecross MI. Studies of cotyledon protoplast cultures from *B. napus*, *B. campestris* and *B. oleracea* II: Callus formation and plant regeneration. Plant Cell Tissue Organ Cult. 1995;40:73–84.

[20] Sheng X, Zhao Z, Yu H, Wang J, Xiaohui Z, Gu H. Protoplast isolation and plant regeneration of different doubled haploid lines of cauliflower (*Brassica oleracea* var. *botrytis*). Plant Cell Tissue Organ Cult. 2011;107:513–20.

[21] Kiełkowska A, Adamus A. An alginate-layer technique for culture of *Brassica oleracea* L. protoplasts. Vitro Cell Dev Biol Plant J Tissue Cult Assoc. 2012;48:265–73. 10.1007/s11627-012-9431-6 PMC3337407 22593638

[22] Kiełkowska A, Adamus A. Peptide growth factor phytosulfokine-α stimulates cell divisions and enhances regeneration from *B. oleracea* var. *capitata* L. protoplast culture. J Plant Growth Regul. 2019;38:931–44.

[23] Stajič E. Improvements in protoplast isolation protocol and regeneration of different cabbage (*Brassica oleracea* var. *capitata* L.) cultivars. Plants. 2023;12:3074. 37687321 10.3390/plants12173074 PMC10489862

[24] Kaur ND, Vyvadilová M, Klíma M, Bechyně M. A simple procedure for mesophyll protoplast culture and plant regeneration in *Brassica oleracea* L. and *Brassica napus* L. Czech J Genet Plant Breed. 2006;42:103–10.

[25] Kirti PB, Bhat SR, Kumar VD, Prakash S, Chopra VL. A simple protocol for regenerating mesophyll protoplasts of vegetable *Brassicas*. J Plant Biochem Biotechnol. 2001;10:49–51.

[26] Hussain M, Li H, Badri Anarjan M, Lee S. Development of a general protoplast-mediated regeneration protocol for *Brassica*: cabbage and cauliflower as examples. Hortic Environ Biotechnol. 2024;65:313–21.

[27] Pauk J, Fekete S, Vilkki J, Pulli S. Protoplast culture and plant regeneration of different agronomically important *Brassica* species and varieties. Agric Food Sci. 1991;63:371–8.

[28] Murashige T, Skoog F. A revised medium for rapid growth and bio assays with tobacco tissue cultures. Physiol Plant. 1962;15:473–97.

[29] Chen LP, Zhang MF, Xiao QB, Wu JG, Hirata Y. Plant regeneration from hypocotyl protoplasts of red cabbage (*Brassica oleracea*) by using nurse cultures. Plant Cell Tissue Organ Cult. 2004;77:133–8.

[30] Menczel L, Nagy F, Kiss ZR, Maliga P. Streptomycin resistant and sensitive somatic hybrids of *Nicotiana tabacum + **Nicotiana knightiana*: correlation of resistance to *N. tabacum* plastids. Theor Appl Genet. 1981;59:191–5. 24276446 10.1007/BF00264975

[31] Kao KN, Michayluk MR. Nutritional requirements for growth of *Vicia hajastana* cells and protoplasts at a very low population density in liquid media. Planta. 1975;126:105–10. 24430152 10.1007/BF00380613

[32] Gamborg OL, Miller RA, Ojima K. Nutrient requirements of suspension cultures of soybean root cells. Exp Cell Res. 1968;50:151–8. 5650857 10.1016/0014-4827(68)90403-5

[33] Pelletier G, Primard C, Vedel F, Chetrit P, Remy R, Rousselle, et al. Intergeneric cytoplasmic hybridization in *Cruciferae* by protoplast fusion. Mol Gen Genet. 1983;191:244–50.

[34] Nakayama Y, Kusano M, Kobayashi M, Manabe R, Watanabe M. Catabolic reprogramming of *Brassica rapa* leaf mesophyll protoplasts during the isolation procedure. Plant Growth Regul. 2023;99:337–57.

[35] Kiełkowska A, Adamus A. Exogenously applied polyamines reduce reactive oxygen species, enhancing cell division and the shoot regeneration from *Brassica oleracea* L. var. *capitata* protoplasts. Agronomy. 2021;11:735.

[36] Eeckhaut T, Lakshmanan PS, Deryckere D, Van Bockstaele E, Van Huylenbroeck J. Progress in plant protoplast research. Planta. 2013;238:991–1003. 23955146 10.1007/s00425-013-1936-7

[37] Jie EY, Kim SW, Jang HR, In DS, Liu J-R. Myo-inositol increases the plating efficiency of protoplast derived from cotyledon of cabbage (*Brassica oleracea* var. *capitata*). J Plant Biotechnol. 2011;38:69–76.

[38] Sahab S, Hayden MJ, Mason J, Spangenberg G. Mesophyll protoplasts and PEG-mediated transfections: transient assays and generation of stable transgenic canola plants. In: Barone P, Smith M, editors. Kumar S. Transgenic Plants. Methods in Molecular Biology. New York: Humana Press; 2019. p. 131–52. 10.1007/978-1-4939-8778-8_10 30415334

[39] Davey MR, Anthony P, Power JB, Lowe KC. Plant protoplast technology: Current status. Acta Physiol Plant. 2005;27:117–30.

[40] Deryckere D, Eeckhaut T, Van Huylenbroeck J, Van Bockstaele E. Low melting point agarose beads as a standard method for plantlet regeneration from protoplasts within the *Cichorium* genus. Plant Cell Rep. 2012;31:2261–9. 22926032 10.1007/s00299-012-1335-8

[41] Nagata T, Takebe I. Plating of isolated tobacco mesophyll protoplasts on agar medium. Planta. 1971;99:12–20. 24487444 10.1007/BF00392116

[42] Shillito RD, Paszkowski J, Potrykus I. Agarose plating and a bead type culture technique enable and stimulate development of protoplast-derived colonies in a number of plant species. Plant Cell Rep. 1983;2:244–7. 24258119 10.1007/BF00269151

[43] Brodelius P, Nilsson K. Permeabilization of immobilized plant cells, resulting in release of intracellularly stored products with preserved cell viability. Eur J Appl Microbiol Biotechnol. 1983;17:275–80.

[44] Jones AMP, Shukla MR, Biswas GC, Saxena PK. Protoplast-to-plant regeneration of *American elm* (*Ulmus americana*). Protoplasma. 2015;252:925–31. 25359187 10.1007/s00709-014-0724-y

[45] Grzebelus E, Szklarczyk M, Greń J, Śniegowska K, Jopek M, Kacińska I, et al. Phytosulfokine stimulates cell divisions in sugar beet (*Beta vulgaris* L.) mesophyll protoplast cultures. Plant Growth Regul. 2012;67:93–100.

[46] Eeckhaut T, Van Houtven W, Bruznican S, Leus L, Van Huylenbroeck J. Somaclonal variation in Chrysanthemum × morifolium protoplast regenerants. Front Plant Sci. 2020;11:2104. 10.3389/fpls.2020.607171 PMC7775395 33391318

[47] Stelmach K, Grzebelus E. Plant regeneration from protoplasts of *Pastinaca sativa* L. via somatic embryogenesis. Plant Cell Tissue Organ Cult. 2023;153:205–17.

[48] Jeong YY, Lee HY, Kim SW, Noh YS, Seo PJ. Optimization of protoplast regeneration in the model plant *Arabidopsis thaliana*. Plant Methods. 2021;17:21. 33622383 10.1186/s13007-021-00720-x PMC7901198

[49] Kiełkowska A, Adamus A. Embedding in filter-sterilized alginate enhances *Brassica oleracea* L. protoplast culture. Acta Biol Cracoviensia Bot. 2014;56:2.

[50] Davey MR, Anthony P, Power JB, Lowe KC. Plant protoplasts: Status and biotechnological perspectives. Biotechnol Adv. 2005;23:131–71. 15694124 10.1016/j.biotechadv.2004.09.008

[51] Glimelius K. High growth rate and regeneration capacity of hypocotyl protoplasts in some *Brassicaceae*. Physiol Plant. 1984;61:38–44.

[52] Kiełkowska A, Adamus A. Early studies on the effect of peptide growth factor phytosulfokine-α on *Brassica oleracea* var. *capitata* L. protoplasts. Acta Soc Bot Pol. 2017;86:3558.

[53] Vaughn KC, Duke SO. Function of polyphenol oxidase in higher plants. Physiol Plant. 1984;60:106–12.

[54] Saxena PK, Gill R. Removal of browning and growth enhancement by polyvinylpolypyrrolidone in protoplast cultures of *Cyamopsis tetragonoloba* L. Biol Plant. 1986;28:313–5.

[55] Dietert MF, Barron SA, Yoder OC. Effects of genotype on *in vitro* culture in the genus *Brassica*. Plant Sci Lett. 1982;26:233–40.

[56] Holme IB, Torp AM, Hansen LN, Andersen SB. Quantitative trait loci affecting plant regeneration from protoplasts of *Brassica oleracea*. Theor Appl Genet. 2004;108:1513–20. 14740090 10.1007/s00122-003-1570-z

[57] Li X, Sandgrind S, Moss O, Guan R, Ivarson E, Wang ES, et al. Efficient protoplast regeneration protocol and CRISPR/Cas9-mediated editing of glucosinolate transporter (gtr) genes in rapeseed (*Brassica napus* L.). Front Plant Sci. 2021;12:680859. 34305978 10.3389/fpls.2021.680859 PMC8294089

[58] Wu LY, Shang GD, Wang FX, Gao J, Wan MC, Xu ZG, et al. Dynamic chromatin state profiling reveals regulatory roles of auxin and cytokinin in shoot regeneration. Dev Cell. 2022;57:526–42. 35063083 10.1016/j.devcel.2021.12.019

[59] Schraudolf H, Reinert J. Interaction of plant growth regulators in regeneration processes. Nature. 1959;184:465–6.

[60] Khan UM, Shaheen N, Farooq A, Maqbool R, Khan SH, Azhar MT, et al. Optimization of regeneration and *Agrobacterium*-mediated transformation protocols for bi and multilocular varieties of *Brassica rapa*. Plants. 2023;12:161 10.3390/plants12010161 PMC9824786 36616290

[61] Al-Khayri JM, Huang FH, Morelock TE, Busharar TA. Stimulation of shoot regeneration in spinach callus by gibberellic acid. HortScience. 1992;27:1046–1046.

[62] Ganesan M, Jayabalan N. Carbon source dependent somatic embryogenesis and plant regeneration in cotton, *Gossypium hirsutum* L. cv. SVPR2 through suspension cultures. Indian J Exp Biol. 2005;43:921–5. 16235728

[63] Hossain MJ, Bari MA, Ara NA, Islam SS. Effect of carbon sources on cell growth and regeneration ability in three cultivars of banana. J Bio-Sci. 2009;17:83–8.

[64] Chutipaijit S, Sutjaritvorakul T. Improvement of plant regeneration frequency from carbon sources in aromatic rice (*Oryza sativa* L.). Iran J Sci Technol Trans Sci. 2018;42:1131–7.

[65] Kumar GP, Subiramani S, Govindarajan S, Sadasivam V, Manickam V, Mogilicherla K, et al. Evaluation of different carbon sources for high frequency callus culture with reduced phenolic secretion in cotton (*Gossypium hirsutum* L.) cv. SVPR-2. Biotechnol Rep. 2015;7:72–80. 10.1016/j.btre.2015.05.005 PMC5466046 28626717

[66] Yaseen M, Ahmad T, Sablok G, Standardi A, Hafiz IA. Review: role of carbon sources for *in vitro* plant growth and development. Mol Biol Rep. 2013;40:2837–49. 23212616 10.1007/s11033-012-2299-z

[67] Ahmad T, Abbasi NA, Hafiz IA, Ali A. Comparison of sucrose and sorbitol as main carbon energy sources in micropropagation of peach rootstock GF-677. Pak J Bot. 2007;39:4.

[68] Vítová L, Stodůlková E, Bartoníčková A, Lipavská H. Mannitol utilisation by celery (*Apium graveolens*) plants grown under different conditions *in vitro*. Plant Sci. 2002;163:907–16.

[69] de Paiva Neto VB, Otoni WC. Carbon sources and their osmotic potential in plant tissue culture: does it matter? Sci Hortic. 2003;97:193–202.

[70] Pogrebnyak N, Markley K, Smirnov Y, Brodzik R, Bandurska K, Koprowski H, et al. Collard and cauliflower as a base for production of recombinant antigens. Plant Sci. 2006;171:677–85.

